# Multiple Fungicide-Driven Alterations in Azole-Resistant *Aspergillus fumigatus*, Colombia, 2015

**DOI:** 10.3201/eid2201.150978

**Published:** 2016-01

**Authors:** Patrice Le Pape, Rose-Anne Lavergne, Florent Morio, Carlos Alvarez-Moreno

**Affiliations:** Université de Nantes, Nantes, France (P. Le Pape, R.-A. Lavergne, F. Morio);; Universidad Nacional de Colombia and Clinica Universitoria Colombia, Bogota, Colombia (C. Alvarez-Moreno)

**Keywords:** *Aspergillus fumigatus*, Colombia, triazoles, antimicrobial resistance, fungal, aspergillosis, fungi, fungicide

**To the Editor:** We read with interest the report by van der Linden et al. about the prevalence of azole-resistant *Aspergillus fumigatus* isolates from 19 countries, including 2 from the Americas (Brazil and the United States) (*1*). Recent reports have suggested a link between use of fungicides in agricultural practices and the presence of triazole-resistant *A. fumigatus* among azole-naive persons (*2*). These resistant strains harbored the TR34/L98H and TR46/Y121F/T289A mutations in the *CYP51A* gene and its promoter region. These novel mechanisms of resistance have been reported both in environmental and clinical samples in Europe, Asia, and Africa, suggesting a broad geographic spread. However, clinical isolates from 22 states in the United States (*3*) and a few isolates from Latin America (*1*,*4*) failed to show any fungicide-driven resistance in *A. fumigatus* in these continents, even though use of pesticides is a widespread practice in the Americas. Colombia was ranked fourth in the world in 2010 for the use of pesticides, reportedly using 14.5 tons/1,000 ha, 30% of which were fungicides (*5*). Among the fungicides approved by Colombia’s regulatory agency, the Colombian Agricultural Institute (*6*), tebuconazole and difenoconazole are largely used in the flower industry, more specifically in Cundinamarca, where 60% of Colombia’s flowers are produced.

In 2015, we conducted a study for which 60 soil samples from flower fields and greenhouses were collected in the outskirts of Bogota, Cundinamarca. Samples were inoculated on Sabouraud agar at 43°C, and positive samples were screened for azole-resistance on agar supplemented with either itraconazole (4 mg/L) or voriconazole (4 mg/L). Of the 38 resistant *Aspergillus* strains, 20 were selected (up to 5 colonies for each positive culture), identified as *A. fumigatus* by β-tubulin gene sequencing, and analyzed for *CYP51A* gene alterations (*7*). Results showed great diversity in molecular resistance with the presence of TR46/Y121F/T289A (n = 17), TR34/L98H (n = 1), and TR53 (n = 1) mutations; 1 isolate had a wild-type *CYP51* sequence (*8*).

Our study highlights the presence of *A. fumigatus* harboring fungicide-driven alterations in Colombia, South America. The results indicate the importance of initiating active agricultural surveillance along with close monitoring of drug resistance in clinical isolates from naive and azole-exposed patients in these countries. Clinical management of *Aspergillus* disease can be challenging because of unfavorable clinical outcomes after patients have acquired multi-azole–resistant strains from the environment (*9*). Additional studies are needed to evaluate the extent to which pesticide use in floriculture and agriculture (e.g., coffee and banana) contributes to azole resistance in Colombia.

## References

[1] van der Linden JWM, Arendrup MC, Warris A, Lagrou K, Pelloux H, Hauser PM, Prospective multicenter international surveillance of azole resistance in *Aspergillus fumigatus.* Emerg Infect Dis. 2015;21:1041–4. 10.3201/eid2106.140717 25988348 PMC4451897

[2] Snelders E, van der Lee HAL, Kuijpers J, Rijs AJMM, Varga J, Samson RA. Emergence of azole resistance in Aspergillus fumigatus and spread of a single resistance mechanism. PLoS Med. 2008;5:e219 . 10.1371/journal.pmed.0050219 18998768 PMC2581623

[3] Pham CD, Reiss E, Hagen F, Meis JF, Lockhart SR. Passive surveillance for azole-resistant *Aspergillus fumigatus*, United States, 2011–2013. Emerg Infect Dis. 2014;20:1498–503. 10.3201/eid2009.140142 25148217 PMC4178384

[4] Lockhart SR, Frade JP, Etienne KA, Pfaller MA, Diekema DJ, Balajee SA. Azole resistance in *Aspergillus fumigatus* isolates from the ARTEMIS global surveillance study is primarily due to the TR/L98H mutation in the cyp51A gene. Antimicrob Agents Chemother. 2011;55:4465–8. 10.1128/AAC.00185-11 21690285 PMC3165364

[5] Food and Agriculture Organization of the United Nations Statistics Division. FAOSTAT/agri-environmental indicators/pesticides. Rome: The Organization; 2015 [cited 2015 May 25]. http://faostat3.fao.org/download/E/EP/E

[6] Instituto Colombiano Agropecuario. Registros de venta de plaguicidas químicos de uso agricola—Septiembre 30 De 2015. Bogata (Colombia): The Institute; 2015 [cited 2015 Feb 12]. http://www.ica.gov.co/getdoc/2dae6093-c021-49d1-8b29-c9dfebce2757/REGISTROS-DE-VENTA–PQA-24-01-09.aspx

[7] Lavergne RA, Morio F, Favennec L, Dominique S, Meis JF, Gargala G, First description of azole-resistant *Aspergillus fumigatus* due to TR46/Y121F/T289A mutation in France. Antimicrob Agents Chemother. 2015;59:4331–5. 10.1128/AAC.00127-15 25918139 PMC4468656

[8] Hodiamont CJ, Dolman KM, Ten Berge IJ, Melchers WJ, Verweij PE, Pajkrt D. Multiple-azole-resistant *Aspergillus fumigatus* osteomyelitis in a patient with chronic granulomatous disease successfully treated with long-term oral posaconazole and surgery. Med Mycol. 2009;47:217–20. 10.1080/13693780802545600 19101840

[9] Chowdhary A, Kathuria S, Xu J, Meis JF. Emergence of azole-resistant *Aspergillus fumigatus* strains due to agricultural azole use creates an increasing threat to human health. PLoS Pathog. 2013;9:e1003633. 10.1371/journal.ppat.1003633 24204249 PMC3812019

